# Imaging in Patients with Merkel Cell Carcinoma

**DOI:** 10.1155/2013/973123

**Published:** 2013-02-06

**Authors:** Elisabeth Enzenhofer, Philipp Ubl, Christian Czerny, Boban M. Erovic

**Affiliations:** Department of Otolaryngology Head and Neck Surgery, Medical University of Vienna, Waehringer Guertel 18-20, 1090 Vienna, Austria

## Abstract

Merkel cell carcinoma (MCC) is a rare, aggressive neuroendocrine tumor of the skin with a mortality rate of approximately 25% (Peloschek et al., 2010). Accurate assessment of nodal involvement in patients with MCC predicts significantly overall outcome (Smith et al., 2012 and Ortin-Perez et al., 2007). Due to the rarity of this highly aggressive disease, only a few imaging reports on MCC were published, and subsequently still to date no accepted imaging algorithm for MCC is available. For primary staging of MCC, general recommendations have included ultrasonography, chest X-ray CT, and MRI, but recent articles show that the use of sentinel node and FDG-PET/PET-CT is gaining more and more importance.

## 1. Introduction and Overview

Merkel cell carcinoma is a rare and highly aggressive neuroendocrine tumor of the skin. It develops predominantly on sun-exposed area of the head and neck [1, 2]. In 1972, Toker described an unknown, trabecular carcinoma of the skin in five caucasian patients [3]. In 1982, Tang and Toker proposed that the MCC derives from the Merkel cell, a mechanoreceptor of the hair follicle [3, 4].

MCC typically develops rapidly and manifests as firm, nontender, dome-shaped red, purple or violet nodule [5, 6]. The overlying skin is smooth and shiny, sometimes exhibiting ulcerative, acneiform, or telangiectatic features [5, 7]. 

MCC tends to metastasize to the regional nodes and in 50% of the patients it spreads hematogenously to other organs [8], that is, the liver, bone, brain, and lung [2]. In 1993, Haag and colleagues defined a commonly used staging system [2, 9]: stage I is defined by local disease without lymph node involvement or distant metastases, in stage II carcinoma has spread to lymph nodes but no systemic metastases are detectable, and in stage III distant metastases are detectable [9].

Diagnosis of MCC can be challenging because in many cases MCC lesions mimic benign skin lesions [10]. Unfortunately, in clinical practice, lesions highly suspicious for Merkel cell carcinoma are often biopsied or nonaccurately resected with close margins [2]. In fact, patients presenting with unclear new skin lesions should undergo clinical examination, and lesion still highly suspicious for Merkel cell carcinoma should be excised with clear and wide margins. Diagnosis and management of nodal metastasis in patients without a primary tumor can be challenging. In particular MCC metastasis can mimic metastasis from other small cell neoplasms, that is, for example lung carcinoma [3, 11]. In 2%–19% of the patients no primary tumor can be found—defined as MCC of unknown primary (MCCUP) [11]. Due to the rarity of this disease, the literature on MCCUP is very limited [12, 13].

Agelli performed multiple epidemiological studies showing that between 1986 and 2001 the age-adapted incidence of MCC has increased 3-fold with an annual increase of 8% [14]. This growing incidence rate has given a significant input for a growing interest in disease management of patients with Merkel cell carcinoma [15, 16]. 

Unfortunately, there is sparse literature on imaging algorithms in patients with Merkel cell carcinoma and no widely accepted guidelines for imaging of Merkel cell carcinoma are available [5, 8]. This paper reviews the literature on imaging of Merkel cell carcinoma discussing the role of the most recent imaging and diagnostic tools. 

## 2. Ultrasonography

Ultrasonography is a highly accurate and cost-effective technique in tumor staging. In regard to Merkel cell carcinoma, work up and staging of the neck should be started with an ultrasonographic examination [2]. 

Primary skin lesions can appear as single or multicentric hypoechoic solid nodules arising from the dermis and extending in the subcutaneous fat, with variable degrees of posterior acoustic transmission [5, 8]. Ultrasonographic features of Merkel cell carcinoma appear similar to more common skin tumors such as melanoma or basal cell carcinoma [8]. It has been shown that in sonographically easy accessible regions, such as the neck, differentiation of malignant from benign lymph nodes can be achieved with an accuracy of 89%–94% [17, 18]. Furthermore, ultrasonography has a key role in real-time imaging during fine needle biopsy of nonpalpable lesions of Merkel cell carcinoma [8]. Except for a few published case reports, ultrasound guided and nonguided fine needle aspiration biopsy has been rarely described in MCC patients [19–23]. Definitive diagnosis of metastatic disease is challenging with fine needle aspiration cytology alone [20]. The cytomorphology resembles numerous other malignancies such as malignant lymphoma and malignant melanoma [23]. Nevertheless, FNA of MCC can provide an accurate and reliable diagnosis of primary or recurrent metastatic lesions [23]. In patients where positive nodes are proven, a full body imaging should be done to detect distant metastases [16]. 

## 3. Sentinel Node Biopsy (SLNB)

Sentinel lymph node biopsy provides the unique capacity to detect metastasis and micrometastasis and subsequently lymph metastasis node draining [5] in patients with melanoma [5], squamous cell carcinoma [24], and MCC [5] by using lymphoscintigraphy [25]. SLNB in patients with Merkel cell carcinoma appears to be a reliable staging technique, whereas the prognostic relevance of positive tumor status of the sentinel node still remains unclear [26]. 

In up to two-thirds of patients with stage I MCC disease, regional nodal spread has been diagnosed at initial presentation with SNLB, and in only 7%−31% nodes are clinically palpable in patients with stage II disease [5]. 

Lymphatic drainage pathways in the head and neck region are more variable than in any other location of the body and are challenging to be accurately predicted [27]. Occasionally, head and neck lymphoscintigrams fail to identify a definitive lymphatic drainage pattern [27]. In particular, unexpected nodal drainage is seen in 37%–84% of cases and is often missed without the use of lymphoscintigraphic guidance [5, 28]. Negative sentinel biopsy appears to be a relevant prognostic factor for disease-free survival [26]. Consequently, false-negative findings in lymphadenectomy are leading to inadequate staging of MCC and aggressive but unnecessary complete nodal dissection in patients with true stage I disease [5]. 

However, Stadelmann and colleagues showed that in 5%–6,8% of patients with melanoma or Merkel cell carcinoma of the head and neck region, no nodal disease could be detected [27]. In particular, in 5 out of 74 clinically node-negative patients who underwent preoperative lymphoscintigraphy, lymphoscintigram failed to identify positive nodes metastases [27]. In 2002, Nguyen and colleagues recommended lymphoscintigraphy in combination with perioperative lymphatic mapping.

## 4. Computed Tomography (CT)

Due to the usefulness of CT for imaging lymph nodes of the head and neck as well as for nodular metastases in subcutaneous fat and visceral metastases, several authors proposed that CT is a reliable imaging method for the initial staging of patients with Merkel cell carcinoma [2, 5, 8]. In particular, Colgan and colleagues proposed sensitivity and specificity rates of 47% and 97%, respectively, with positive and negative predictive values of 94% and 68%, respectively, for diagnosis of lymph node involvement by CT imaging [29]. However, Peloschek and coworkers claimed a specificity of 96.2% and a sensitivity of 89.1% for CT in diagnostic imaging of Merkel cell carcinoma including lymph node involvement as well as evaluation of distant metastasis [2].

Compared to the muscle, primary skin lesions appear as isodense to slightly hyperdense cutaneous rounded nodules extending below the skin [30]. Cutaneous fat stranding adjacent to the primary lesion suggests engorgement and edema from lymphatic invasion [8]. Furthermore, enhanced CT scan is able to demonstrate high-attenuation lymphadenopathy and soft CT scan is able to demonstrate high-attenuation tissue nodules, which are often clinically silent [5, 8, 30], suggesting focal metastases [30]. Lymphadenopathy mostly occurs in the neck, especially in the parotid region followed by the axilla, mediastinum, retroperitoneum, and groin. Distant metastases include local and retroperitoneal lymph nodes, liver, bone, brain, and lung [31]. Using CT-imaging, metastases of abdominal organs manifest as hypervascular lesions with ring-like enhancement [5]. Soft-tissue metastases may involve the chest wall or abdominal wall with musculoskeletal invasion. Gollub and colleagues conducted a study in 12 patients with MCC and showed the ability of CT scanning to detect visceral and nodal metastases. They suggest follow-up CT scans at 3, 6, 12, and 18 months after initial treatment to discover recurrent disease [30].

## 5. Magnetic Resonance Imaging (MRI)

There are only a few studies and case reports describing the usefulness of MRI in patients with MCC. In particular, case reports on large primary tumors of the sinonasal region [32], and abdominal wall [33] described MCC lesions as inhomogeneous in signal intensity on T1- and T2-weighted images [33, 34]. Focal central increased signal intensity on T2-weighted images within large lesions has been described as being associated with histologically proven central necrosis and hemorrhage [33, 34]. In MRI scans, lymphatic satellite lesions are reflected by reticular stranding and subcutaneous masses. The same appearance of satellite lesions can be observed by CT imaging. Large lymph node metastases appear as lesions with fine, compressed, retained fatty tissue [34].

Colgan showed in a study of 7 patients who underwent first MRI followed by sentinel lymph node biopsy or regional lymph node dissection a positive predictive value of 0% and a negative predictive value of 67% for the MRI [29]. However, Anderson and colleagues showed in 15 patients that MRI improves differentiation of distant metastases [34]. Furthermore, intramuscular masses and perifascial tumors were better defined on MRI than by CT imaging [34]. 

MRI in Merkel cell carcinomas is highly accurate for evaluating soft tissue metastases, as well as involvement of brain and bone marrow. Invasion of the central nervous system is rare; however, in case of neurologic symptoms, workup should be performed with MRI [5, 35]. 

## 6. Somatostatin Receptor Scintigraphy (SRS)

The rational for performing somatostatin receptor scintigraphy in MCC patients to detect locoregional and distant metastatic disease is based on the neuroendocrine characteristics of MCC. In 1992, Kweekeboom and colleagues presented data for the effectiveness of SRS in 4 patients with MCC. In all 4 patients, in whom the tumor was detected by CT and sonography, tumor sites were also detected in SRS. They showed that SRS had an equal or greater sensitivity than CT for imaging of MCC [36].

Nevertheless, more recent studies observed a limited sensitivity of SRS as well as a high rate of false positive and negative results [37–39]. Guiltera presented their 7-year experience with 20 patients with MCC. In particular, sensitivity of 78% and specificity of 96% for SRS of Merkel cell carcinoma could be observed [38]. 

A comparison between SRS, CT and MRI showed that tissue SRS is less affected by inflammation, edema, granulation tissue at surgically pretreated or irradiated sites [5]. However, there is a significantly limited value in organs showing a physiological uptake of radiolabelled octreotide such as liver, adrenal glands, pancreas, thyroid gland, and spleen [5, 37]. This causes a low tumor-to-background ratio, which hampers detection of metastasis near organs with a high physiological uptake of the tracer [37]. Further, other systemic diseases such as sarcoidosis, tuberculosis, Wegener's granulomatosis, non-Hodgkin lymphoma, or Hodgkin's disease have also led to false positive SRS results [37, 40].

Unfortunately, a limited use of SRS in diagnostic evaluation of Merkel cell carcinoma. Therefore many authors do not recommend SRS for routine imaging [37, 38]. 

## 7. Positron Emission Tomography (PET) and Positron Emission Computed Tomography (PET-CT)

Within the last years nuclear medicine, especially PET and PET-CT, has gained importance in diagnostic imaging of Merkel cell carcinoma. Since MCC is a rapid growing tumor, it is expected that tumor cells have an increased glycolysis [2].

**Figure 1:**
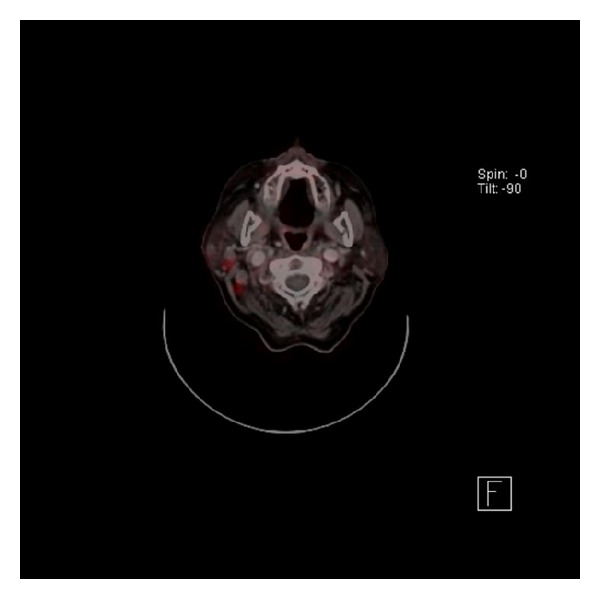
PET/CT: pathologic enhancement in the right parotideal region.

^18^F-FDG is a glucose analog and a surrogate marker for glucose metabolism [41]. In particular, increased glycolysis in certain areas compared to healthy tissue is a distinctive feature of malignant transformation. Increased glycolysis can be captured using the FDG positron emission tomography (PET) technique allowing differentiation between normal and malignant tissue [5] as shown in Figure 1. 

The main difficulty with PET alone is the lack of an anatomical reference frame. The hybrid of FDG-PET and the morphological data of CT have potential to improve specificity of PET [2].

Several studies in ^18^FDG-PET and PET-CT supported the effectiveness in detecting locoregional nodal and distant metastatic disease and subsequently staging in patients with MCC [2, 42–47].

Unfortunately, only few data are available comparing ^18^FDG-PET and PET-CT with the gold standard of histopathologic nodal evaluation and other imaging tools in MCC patients.

In a study comparing FDG-PET-CT, MRI, bone scan, and computerized tomography in 11 patients, the authors could show that FDG-PET has a sensitivity of 92% and specificity of 100%, and in 3 patients FDG-PET-CT allowed a more precise anatomic localization of lesions [42]. Furthermore, Concannon et al. found, in a retrospective study of 18 patients with MCC who underwent FDG-PET-CT imaging, that FDG-PET-CT resulted in altered staging in 33% of patients and in changes in disease management in 43% of the patients [36]. However, a retrospective study in 15 patients showed a significant advantage of FDG-PET-CT compared to clinical examination in 46% of patients, whereas sensitivity, specificity, and positive and negative predictive value were the same for PET-CT and CT, respectively [48].

In a retrospective study, Peloschek and colleagues described that FDG-PET has a sensitivity of 85,7% and a specificity of 96,2% compared to a sensitivity of 95,5% and specificity of 89,1% for conventional imaging methods [2]. In another study, Grewal et al. reported the sensitivity and specificity of FDG-PET in MCC as 79% to 92% [49].

The most significant drawback of this technique is the fact that in some cases metabolic trapping can be nonspecific and in addition to tumor cells it can also be found in sites of inflammation or infection [50]. In case of brain metastases FDG-PET scanning is significantly hampered due to the high metabolic rate. Subsequently high cerebral background impairs detection of metastatic lesions in the brain [51]. Furthermore, some authors describe a failure of FDG-PET-CT in detection of lymph nodes micrometastases and distant metastatic disease [29, 48].

### 7.1. Alternative Tracers

Biogenic amines are enhanced and accumulated in neuroendocrine tumors and are an alternative PET tracer for MCC to visualize malignant tissue [2]. A case report described that, due to the less intense uptake of ^18^F-DOPA, it is more accurate in diagnosis of brain metastases ^18^F-DOPA compared to FDG-PET and is as accurate in detection of more extracranial metastases [45]. However, Peloschek et al. showed in a study, superior value of FDG-PET in detection of malignant sites of MCC, showing two false negative regions in ^18^F-DOPA-PET [2]. Furthermore, diffuse ^18^F-DOPA uptake was ^18^F-DOPA seen in a region pretreated with surgery and ^18^F-DOPA irradiation, which was similar to that in FDG-PET that hampers the idea of a benefit of ^18^F-DOPA. Thus, ^18^F-DOPA-PET cannot be recommended for diagnostic imaging ^18^F-DOPA in Merkel cell carcinoma [2]. 

### 7.2. Follow-Up Imaging

After treatment of primary lesions of MCC, a close monitoring is required.

For follow-up imaging, we would suggest a routine chest X-ray as well as a computed tomography of the head and neck region 3 months after therapy. Every year after therapy, a chest X-ray, CT and MRI of the head and neck region are recommended. 6, 9, 15, 18, 21, and 30 months after therapy a cervical ultrasonography and a chest X-ray should be performed. Because of the low cost of sonography, it has a high value in routine follow-up imaging of Merkel cell carcinoma [2]. Chest X-ray is a routine imaging technique to evaluate possible pulmonary involvement. Peloschek et al. recommend repetition of FDG-PET 3 months and 1 year after treatment [2]. 

## 8. Discussion

The key task of imaging in patients with Merkel cell carcinoma is staging at the initial presentation and post-therapeutical.

Early recommendations for imaging in MCC included ultrasonography CT, MRI, and octreotide scans [29]. Recently, ^18^FDG-PET has become a valuable and useful imaging technique for staging in patients suffering from MCC. Its diagnostic value is comparable to conventional imaging methods that have a restricted field of view [2]. 

Peloschek et al. recommend that initial staging workup should be started with ultrasonography as it is cost-effective and an accurate imaging method in easy accessible lymph node regions such as the head and neck [2]. There is rarely literature available dealing with ultrasound-guided fine needle biopsy of Merkel cell carcinoma. Definitive diagnosis is difficult but possible and accurate with FNA [19, 20].

In oncologic patients with suspected distant metastases FDG-PET, CT or MRI imaging should be performed. Somatostatin receptor scintigraphy is no longer recommended for routine imaging of Merkel cell carcinoma, as studies showed a high rate of false-positive or false-negative results in detection of Merkel cell carcinomas and metastatic disease [37].

As Merkel cell carcinoma has a high rate of distant metastasis, PET scan has a particular value in imaging and staging workup. 

MRI has a particular value in assessing soft-tissue involvement, whereas CT is used for imaging of thorax and abdomen. Three months and 1 year after treatment, FDG-PET should be repeated for follow-up imaging. Moreover, fusion of FDG-PET with CT or MRI would improve specificity of PET analysis [2]. 

Colgan et al. reported that the use of FDG-PET when compared with traditional computed tomography is significantly more sensitive and equally specific than FDG-PET alone in evaluation of regional lymph node basins in primary MCC [29].

The role of FDG-PET-CT in management of MCC remains to be a matter of debate. However, PET-CT has been shown to have a potential high impact of staging and management of MCC patients with stage I and II disease [43].

To date, there is still no imaging algorithm for Merkel cell carcinoma. Due to the rarity of Merkel cell carcinoma imaging, findings have been reported only in small trials and case reports. On the basis of the existing literature, we would recommend FDG-PET CT as first line imaging of Merkel cell carcinoma. It is a noninvasive imaging technique that has potential to detect occult lesions bigger than 5–8 mm in minimal diameter [48] that are not detectable by other imaging techniques. We suggest that further diagnostic imaging should be obtained depending on the results of lymph node involvement and distant metastases.

However, in case of negative lymph node involvement, we would recommend sentinel lymph node mapping with subsequently performing an ipsilateral neck dissection to confirm lymph node status histopathologically. In our opinion, due to the low morbidity of a neck dissection, it has a high diagnostic and preventive value.

In summary, Merkel cell carcinoma is a highly aggressive skin cancer with a high rate of metastasis and mortality. Since no imaging guidelines are available, more studies are required to define an evidence-based imaging algorithm.
